# Relationship between Exposure to Household Humidifier Disinfectants and Risk of Lung Injury: A Family-Based Study

**DOI:** 10.1371/journal.pone.0124610

**Published:** 2015-05-15

**Authors:** Dong-Uk Park, Ye-Yong Choi, Jong-Ju Ahn, Heung-Kyu Lim, Sun-Kyung Kim, Hyun-Suk Roh, Hae-Kwan Cheong, Jong-Han Leem, Dong-Hee Koh, Hye-Jung Jung, Kyoung-Mu Lee, Jong-Hyeon Lee, Yong-Hwa Kim, Sin-Ye Lim, Do-Myung Paek, Chae-Man Lim, Soo-Jong Hong

**Affiliations:** 1 Department of Environmental Health, Korea National Open University, Seoul, 110-791, South Korea; 2 Asian Citizen's Center for Environment and Health, Seoul, 110-460, South Korea; 3 Data analytics team, tiny Labs, Seoul, 140-822, South Korea; 4 Department of Social and Preventive Medicine, Sungkyunkwan University School of Medicine, Suwon, 440-746, Korea; 5 Department of Occupational & Environmental Medicine, Inha University Hospital, Incheon, South Korea; 6 Department of Occupational and Environmental Medicine, International St. Mary's Hospital, Catholic Kwandong University, Incheon, 210-701, South Korea; 7 Institute of Environmental Safety and Protection, NeoEnBiz Co., Bucheon, 420-806, South Korea; 8 Korea Institute of Toxicology, Daejeon, 305-343, South Korea; 9 Department of Occupational & Environmental Medicine, College of Medicine, Kyung Hee University, 130-791, Seoul, Korea; 10 School of Public Health, Seoul National University, Seoul, 151-742, South Korea; 11 Department of Pulmonary Medicine and Critical Care Medicine, Asan Medical Center, University of Ulsan College of Medicine, Seoul, South Korea; 12 Department of Pediatrics, Childhood Asthma Atopy Center, Asan Medical Center, University of Ulsan College of Medicine, Seoul, South Korea; University of Rochester Medical Center, UNITED STATES

## Abstract

**Background:**

In South Korea, a cluster of acute lung disease patients included lung injury disease suspected of being caused by the use of humidifier disinfectants. We examined the relationship between humidifier disinfectant exposure and clinically diagnosed humidifier disinfectant-associated lung injury (HDLI) in a family-based study.

**Methods:**

This case-control study included 169 clinically confirmed HDLI cases and 303 family controls who lived with the HDLI patients. A range of information on exposure to humidifier disinfectants was obtained using a structured questionnaire and field investigations. Odds ratios (ORs) and confidence intervals (CIs) were estimated using unconditional logistic regression models that were adjusted for age, sex, presence of a factory within 1 km of residence, and the number of household chemical products used.

**Results:**

HDLI risk increased approximately two-fold or more among the highest quartile compared with the lowest quartile in terms of the hours sleeping in a room with an operating humidifier treated with disinfectant (adjusted OR = 2.0, 95 % CI = 1.1-3.7), average hours of disinfectant-treated humidifier use per day (adjusted OR = 2.1, 95 % CI = 1.0-4.5), airborne disinfectant intensity (adjusted OR = 2.6, 95% CI = 1.2-5.3), and cumulative disinfectant inhalation level (adjusted OR = 2.0, 95% CI = 1.0-4.1). HDLI risk increased as the distance of the bed from humidifier gets shorter; compared with longer distance (> 1 m), the odds ratio was 2.7 for 0.5 to 1 m (95 % CI = 1.5-5.1) and 13.2 for <0.5 m (95 % CI = 2.4-73.0).

**Conclusions:**

The use of household humidifier disinfectants was associated with HDLI risk in a dose-response manner.

## Introduction

Several types of disinfectants were used to prevent microbial contamination in humidifiers in South Korea from 1994, but their use has been banned since 2011 due to the occurrence of a series of cases diagnosed with humidifier disinfectant-associated lung injury (HDLI) [1]. South Korea is believed to be the only country where disinfectants were added to the water in humidifiers for extended periods of time instead of the disinfectants solely being used for cleaning the humidifiers and then discarded. More than ten humidifier disinfectant products featuring several different types of components were marketed in South Korea without toxicological testing for inhalation health effects. The major disinfectants added to humidifiers were polyhexamethylene guanidine phosphate (PHMG, CAS #: 89697-78-9), oligo(2-(2-ethoxy)ethoxyethyl guanidinium (PGH, CAS #: 374572-91-5), chloromethylisothiazolinone (CMIT, CAS #: 26172-55-5), methylisothiazolinone (MIT, CAS #: 2682-20-4). PHMG and PGH, the guanidine chemical group, were not mixed with other components for humidifier disinfectant products, while a mixture of CMIT and MIT, the non-guanidine chemical group, was included in several humidifier disinfectant products. PHMG and PGH are well-known disinfectants for the treatment of various medical conditions. They have been classified as nontoxic crystalline compounds for oral intake or skin application [2]. To our knowledge, there has been no study reporting inhalation health effects due to the use of disinfectants for humidifier.

A number of studies conducted in South Korea have recently concluded that humidifier disinfectants can cause HDLI with diffuse pulmonary fibrosis [3–8]. So far two hospital-based case-control studies have reported that the use of humidifier disinfectant was significantly associated with a cluster of HDLI (unadjusted OR = 2.7, 95% CI = 1.4–5.9) [8] (unadjusted OR = 47.3, 95% CI = 1.4–5.9) [9]. However, no study has yet evaluated whether HDLI risk was associated with specific disinfectant exposure-related characteristics or with quantitative measures of disinfectant use, including duration, intensity, and cumulative exposure to humidifier disinfectants. Here, we report the relationship between humidifier disinfectant exposure-related characteristics and risk of clinically confirmed HDLI in a family-based case-control study to identify the association between quantitative aspects of disinfectant exposure and HDLI risk.

## Methods

### Study Design and Subjects

In order to properly compensate patients with HDLI caused by the use of humidifier disinfectant, the Korea Center for Disease Control and Prevention (KCDC) officially collected information on individuals with lung disease who presumed that their disease was related to humidifier disinfectant use. As many as 374 self-reported victims were registered with KCDC. A total of 303 non-patients from among family members who lived in the same residence as the registered patients were recruited as a control group for comparison of humidifier disinfectant exposure-related characteristics. A non-patient familial group was chosen because they were expected to have less differential recall bias, provide a more appropriate comparison group than population controls, and be recruited at lower cost [10, 11].

The registered lung disease patient cases (n = 374) were clinically examined by a committee consisting of pediatric pulmonologists, adult pulmonologists, radiologists and pathologists to diagnose and confirm HDLI and its severity. The non-patient control group was not clinically examined. In March 2014, KCDC reported that a total of 169 of the registered lung injury patients (45.2%) were clinically confirmed as HDLI patients. Clinically confirmed HDLI cases were determined based on the combination of clinical manifestations and radiologic findings with pathologic findings in patients for whose lung specimen were available [4, 6, 7, 9]. The typical clinical manifestations include dyspnea, tachypnea, and cough without evidence of respiratory infections. Radiologic findings includemultifocal patchy consolidation in subpleural zones of both lower lungs in the early phase, diffuse centrilobular ground glass opacity lesions involving both lungs in the subacute phase, and diffuse homogeneous ground glass opacity in both lungs with presence of pneumomediastinum in the late phase. Other diffuse lung diseases that appeared on CT were excluded based on our clinical definition, including sequelae of previous infection including pulmonary tuberculosis, pneumoconiosis, idiopathic interstitial pneumonitis, and atypical pneumonia.

In total, our study subjects consisted of 169 clinically diagnosed HDLI patient cases and 303 family non-patient controls (Table 1). Although 205 non-confirmed HDLI patients and cases with non-HDLI lung disease were excluded from the analyses, the 103 non-patient family members who lived with these non-confirmed patients remained in the control group. A total of 303 self-reported non-patients family members who were not clinically examined and confirmed are used as control group. The study protocol was approved by the Institutional Review Boards of Seoul National University (42-2013-07-01), and written informed consent to participate in this study was obtained from all participants or from parents or guardians in case of children cases at enrollment.

**Table 1:** Associations with HDLI for confounding factors.

Classification	Cases(n = 169)		Controls(n = 303)				
	n	%	n	%	Adj. OR	95% CI	
Gender							
Male	72	42.6	153	50.5	1.0	Ref	
Female	97	57.4	150	49.5	1.4	0.9	2
Smoking status							
Never	159	94.1	220	72.6	1.0	Ref	
Ever	6	3.6	27	8.9	0.3	0.1	0.8
Current	0	0.0	51	16.8	N.A.		
No. of household products containing chemical used ^a^							
Q1(<3)	31	18.3	55	18.2	1.0	Ref	
Q2(3)	46	27.2	89	29.4	0.8	0.4	1.6
Q3(5)	29	17.2	51	16.8	1.0	0.5	2.0
Q4(6)	63	37.3	108	35.6	1.0	0.5	1.8
Presence of factory within 1 km of residence ^a^							
No	163	96.4	286.0	94.4	1.0	Ref	
Yes	6	3.6	17.0	5.6	0.5	0.2	1.4
Age group ^b^							
>18 years old	36	21.3	226	74.6	1.0	Ref	
7 through 18 years old	4	2.4	12	4.0	3.3	0.7	15.3
≤ 6 years old	103	60.9	56	18.5	19.4	5.4	70.1
Pregnant women	26	15.4	9	3.0	14.6	6.0	35.8

Note: N.A: Not Applicable

^b^ ORs and CIs were estimated by unconditional logistic regression, adjusted for sex, no. of household products containing chemicals, and presence of a factory within 1 km of residence.

### Assessment and Collection of Humidifier Disinfectant Exposure-Related Information

Participants were contacted by mail and asked to complete a residence and work history calendar. During the subsequent home visit, three trained environmental health scientists with doctoral degrees administered a computer-assisted personal interview that included a lifetime occupational history and questions about demographics and tobacco use and conducted home environment investigations. We asked study participants or, in the case of children, their parents or guardians, to complete detailed questionnaires collecting information related to humidifier disinfectant use and potential confounders such as "number of household chemical products used excluding humidifier disinfectants" and "presence of factories within 1 km of residence" by means of open-ended questions, which were confirmed by researchers. We also extracted additional disinfectant exposure variables such as “average hours sleeping in a room with an operating humidifier treated with disinfectant", "number of humidifier disinfectant brands, "type of disinfectant", and "average distance (in meters) of the bed from humidifier" To aid in recall, participants were shown photographic examples of all the humidifier disinfectant products that had been marketed in South Korea.

The overall framework for estimating the cumulative disinfectant use hours, airborne disinfectant intensity and cumulative level of exposure to humidifier disinfectant was described in detail elsewhere [12]. The methods for estimating cumulative disinfectant use hours, airborne disinfectant exposure intensity (*μg*/m^3^) and cumulative level of inhalation exposure to disinfectant based on the collected disinfectant information and home investigation are briefly described here. Cumulative disinfectant use hours were calculated by multiplying together the total years, months per year, weeks per month, days per week, and hours per day during which humidifier disinfectants were actually used. Hours per day includes hours sleeping in a room with a humidifier that contained disinfectants.

Airborne disinfectant exposure intensity was estimated based on disinfectant volume (mℓ) and the frequency with which it was added to the humidifier per day, disinfectant bulk level (*μg*/mℓ), the volume of the room (m^3^) with a humidifier using disinfectants (area x height, m^3^, measured during the home visit), and the degree of ventilation. The bulk level of humidifier disinfectant was identified in the specified disinfectant brand during a government-mandated inspection conducted by the KCDC (*μg*/mℓ) [13]. A room ventilation factor that was arbitrarily determined according to the level of ventilation in the home (Q7): 1 = no or only minor ventilation less than one hour per day; 0.9 = ventilation between one and two hours per day; 0.8 = ventilation longer than two hours per day. The cumulative inhalation exposure levels of each study subject to disinfectants were calculated by multiplying together the quantitative estimates of disinfectant exposure intensity and cumulative disinfectant use hours. All study subjects (n = 472) were assigned by the exposure team to be exposed or not exposed to humidifier disinfectants based on the presence of material evidence, such as receipts for the purchase of humidifier disinfectants, the residual disinfectant, or a photograph from the past showing the use of disinfectants, and based on the subject’s consistency in responses to disinfectant-related questions. All information and data obtained from investigation into the study subjects were examined for further study analysis by an exposure assessment committee consisting of an environmental health scientist, environmental toxicologist, epidemiologist and environmental medicine specialist. All interviews and investigations for humidifier disinfectant exposure assessment were conducted before reports of clinical test results. The exposure assessment team, including interviewers, was blinded to the clinically diagnosed lung disease information of the study subjects.

### Statistical Analysis

Descriptive statistics of humidifier disinfectant exposure-related characteristics and other variables that may influence the development of HDLI were compared between HDLI patient cases and the non-patient family control group. Study subjects were also classified into four groups according to age and pregnancy to examine potential differences in the level of susceptibility or sensitivity [14]: six years old or younger; between six and 18 years of age; pregnant women, and 19 years of age or older excluding pregnant women (reference).

Quantitative humidifier disinfectant exposure-related variables (i.e.; total disinfectant use years, months, and average hours per day, hours sleeping in a room with an operating humidifier treated with disinfectant, cumulative disinfectant use hours and airborne disinfectant exposure intensity and airborne disinfectant exposure level) were classified into quartiles (< 25^th^, 25^th^-50^th^, 51^th^–75^th^ and > 75^th^) based on the distribution of controls in order to aggregate similar exposures. The lowest quartile was used as the reference group. The average distance of the bed from humidifier (≥ 1 m = reference, 0.5–1 m, and < 0.5 m) and the number of disinfectant brands used (1 = reference, 2 and ≥ 3) were arbitrarily categorized. Tests for trends were conducted by modeling ordinal terms for categories of quantitative variables. Qualitative variables including type of humidifier disinfectant (non-guanidine = reference versus guanidine chemical groups) and the presence of a facility generating hazardous chemicals within 1 km of the place of residence (no = reference versus yes) were dichotomized.

These various humidifier disinfectant exposure-related metrics were assessed singly or in combination in order to evaluate the risk of HDLI. Differences in the distribution of descriptive variables and established risk factors between case and control groups were assessed using chi-square tests for categorical variables and analysis of variance for continuous variables. We estimated the odds ratios (ORs) and their 95% confidence intervals (CIs) using unconditional logistic regression. Each disinfectant-related exposure metric was evaluated individually in models that adjusted for potential covariates, including sex, age, smoking status and potential confounders. All statistical analyses were performed using STATA ver. 12 (STATA Corp, College Station, Texas, USA).

## Results

The HDLI patient case and non-patient family control groups were similar with respect to demographic variables and residence environment (Table 1) for the study as a whole. Pre-school children aged less than 7 years old accounted for 60.9% of HDLI cases and 18.5% of the control group. HDLI patients were slightly more likely to be female (57.4%) than male (42.6%). No significant difference between the cases and control with regard to potential confounders such as the number of household chemicals or the presence of a factory within 1 km of residence was found. Both pregnant women (adjusted OR = 1.9, 95% CI = 1.0–3.6) and pre-school children ≤ 6 years old (adjusted OR = 1.9, 95% CI = 1.0–3.6) showed an increased HDLI risk compared with the adult group of older than 18 years of age (Table 1). Overall, the distribution patterns of humidifier disinfectant exposure-related variables for the HDLI patients were higher than those of the control, especially for pregnant women and patients ≤ 6 years old (Table 2). A clear differences in exposure patterns by age group was observed.

**Table 2:** Humidifier disinfectant use characteristics by HDLI case and control and age group.

Classification	≤6 years old				7–18 years old				Pregnant women				>8 years old			
	Cases (n = 103)	%	Controls (n = 56)	%	Cases (n = 4)	%	Controls (n = 12)	%	Cases (n = 26)	%	Controls (n = 9)	%	Cases (n = 36)	%	Controls (n = 226)	%
Total years of use, years																
Q1(≤0.42)	24	23.3	13	23.2	0	0.0	3	25.0	4	15.4	2	22.2	7	19.4	47	20.8
Q2(0.43–2.25)	28	27.2	15	26.8	0	0.0	1	8.3	8	30.8	3	33.3	7	19.4	53	23.5
Q3(2.26–3.25)	28	27.2	13	23.2	3	75.0	3	25.0	4	15.4	1	11.1	4	11.1	57	25.2
Q4(3.26–4)	20	19.4	14	25.0	1	25.0	5	41.7	10	38.5	3	33.3	18	50.0	68	30.1
P-value	0.126				0.371				0.297				0.146			
Average total months of use per year, months																
Q1(≤3)	5	4.9	2	3.6		0.0		0.0	0	0.0	1	11.1	0	0.0	14	6.2
Q2(4–6)	48	46.6	20	35.7	2	50.0	3	25.0	13	50.0	3	33.3	15	41.7	88	38.9
Q3(7–9)	19	18.4	13	23.2	2	50.0	1	8.3	9	34.6	3	33.3	7	19.4	44	19.5
Q4(10–12)	31	30.1	21	37.5	0	0.0	8	66.7	4	15.4	2	22.2	14	38.9	80	35.4
P-value ^a^	0.13				0.218								0.126			
Average total use hours per day, hours																
Q1(≤10)	11	10.7	10	17.9	1	25.0	2	16.7	5	19.2	3	33.3	5	13.9	51	22.6
Q2(11–12)	26	25.2	15	26.8	3	75.0	4	33.3	9	34.6	2	22.2	8	22.2	56	24.8
Q3(13–14)	37	35.9	15	26.8	0	0.0	3	25.0	4	15.4	3	33.3	8	22.2	59	26.1
Q4(15–24)	27	26.2	16	28.6	0	0.0	3	25.0	8	30.8	1	11.1	15	41.7	60	26.5
P-value	0.129				0.26				0.266			0.0	0.133			
Average hours sleeping in a room with an operating humidifier treated with disinfectant per day, hrs																
Q1(≤10)	16	15.5	14	25.0	1	25.0	3	25.0	7	26.9	3	33.3	5	13.9	65	28.8
Q2(10.1–11)	21	20.4	12	21.4	3	75.0	5	41.7	4	15.4	2	22.2	7	19.4	48	21.2
Q3(11.1–12)	64	62.1	30	53.6	0	0.0	4	33.3	15	57.7	4	44.4	24	66.7	113	50.0
P-value	0.143				0.36				0.312				0.118			
Type of disinfectant																
Non-Guanidine	2	1.9	3	5.4	2	50.0	0	0.0	0	0.0	1	11.1	1	2.8	27	11.9
Guanidine	99	96.1	51	91.1	2	50.0	11	91.7	26	100.0	8	88.9	34	94.4	194	85.8
P-value	0.354				0.012				0.085				0.298			
No of disinfectant products used																
1	59	57.3	33	58.9	0	0.0	5	41.7	16	61.5	5	55.6	20	55.6	136	60.2
2	33	32.0	17	30.4	3	75.0	3	25.0	8	30.8	3	33.3	9	25.0	67	29.6
≥ 3	9	8.7	4	7.1	1	25.0	3	25.0	2	7.7	1	11.1	6	16.7	17	7.5
P-value	0.158				0.326				0.352				0.168			
Average distance of the bed apart from humidifier																
> 1 m	65	63.1	52	92.9	1	25.0	10	83.3	19	73.1	8	88.9	29	80.6	186	82.3
0.5–1 m	31	30.1	4	7.1	3	75.0	1	8.3	5	19.2	0	0.0	3	8.3	37	16.4
< 0.5	4	3.9	0	0.0	0	0.0	1	8.3	2	7.7	1	11.1	4	11.1	2	0.9
P-value	<0.0001				0.028				0.361				<0.0001			
Cumulative use hours, hours ^a^																
Q1(<1260)	22	21.4	16	28.6	1	25.0	3	25.0	5	19.2	3	33.3	4	11.1	62	27.4
Q2(1261–2352)	33	32.0	10	17.9	2	50.0	2	16.7	5	19.2	2	22.2	6	16.7	56	24.8
Q3(2353–5040)	23	22.3	16	28.6	1	25.0	2	16.7	8	30.8	2	22.2	11	30.6	52	23.0
Q4(5041–39116)	22	21.4	14	25.0	0	0.0	5	41.7	8	30.8	2	22.2	15	41.7	54	23.9
P-value	0.24				0.375				0.28				0.036			
Airborne disinfectant intensity, *μg*/m^3^ ^b^																
Q1(<317.1)	18	17.5	12	21.4	2	50.0	2	16.7	9	34.6	4	44.4	6	16.7	55	24.3
Q2(317.2–508.5)	25	24.3	15	26.8	1	25.0	1	8.3	6	23.1	2	22.2	5	13.9	61	27.0
Q3(508.6–942.5)	28	27.2	16	28.6	0	0.0	6	50.0	5	19.2	3	33.3	11	30.6	43	19.0
Q4(942.6–4946.9)	30	29.1	10	17.9	1	25.0	2	16.7	6	23.1	0	0.0	13	36.1	51	22.6
P-value	0.526				0.27				0.418				0.074			

Note: HDLI: Humidifier disinfectant-associated lung injury.

^a^ Cumulative disinfectant use hours were calculated by multiplying together the total years, months per year, weeks per month, days per week, and hours per day.

^b^ Airborne disinfectant intensity was calculated by multiplying together the bulk level of humidifier disinfectant identified in the specified disinfectant brand (*μg*/mℓ), the average volume (mℓ) added to humidifier, and the room volume (area x height, m^3^).

The association between HDLI risk and various exposure metrics describing humidifier disinfectant use were examined (Table 3). HDLI risks were found to significantly increase, approximately two-fold or more, in the highest compared with the lowest quartile of several exposure metrics. These included hours sleeping in a room with an operating humidifier treated with disinfectant (adjusted OR = 2.0, 95% CI = 1.1–3.7), average hours disinfectant-treated humidifier disinfectant used per day (adjusted OR = 2.1, 95% CI = 1.0–4.5) and average distance of the bed from humidifier (adjusted OR = 13.2, 95% CI = 2.4–73.0). We also found that airborne disinfectant exposure intensity (adjusted OR = 2.6, 95% CI = 1.2–5.3) and cumulative disinfectant exposure level (adjusted OR = 2.0, 95% CI = 1.0–4.0) significantly increased the risk of HDLI. These metrics also showed significant *P* trends across exposure categories.

**Table 3:** Association between humidifier disinfectant exposure-related characteristics and HDLI risk.

Classification	Cases (n = 169)		Controls (n = 303)					
	n	%	n	%	Adj. OR	95% CI		P-trend
Total years of use, years								
Q1(<0.42)	35	20.7	65	21.5	1.0	Ref		
Q2(0.43–2.25)	43	25.4	72	23.8	1.0	0.5	2.1	
Q3(2.26–3.25)	39	23.1	74	24.4	0.9	0.5	1.9	
Q4(3.26–4)	49	29.0	90	29.7	1.1	0.6	2.2	0.64
N.I.	3	1.8	2	0.7				
Average total months of use per year, months								
Q1(<3)	5	3.0	17	5.6	1.0	Ref		
Q2(4–6)	78	46.2	114	37.6	3.0	0.9	10.4	
Q3(7–9)	37	21.9	61	20.1	2.3	0.6	8.3	
Q4(10–12)	49	29.0	111	36.6	1.7	0.5	6.0	0.25
Average total use hours per day, hours								
Q1(<10)	22	13.0	66	21.8	1.0	Ref		
Q2(11–12)	46	27.2	77	25.4	1.6	0.7	3.4	
Q3(13–14)	49	29.0	80	26.4	1.6	0.8	3.5	
Q4(15–24)	50	29.6	80	26.4	2.1	1.0	4.5	0.067
N.I.	2	1.2						
Average hours sleeping in a room with an operating humidifier treated with disinfectant per day, hours								
Q1(<10)	29	17.2	79	26.1	1.0	Ref		
Q2(10.1–11)	35	20.7	63	20.8	1.7	0.8	3.5	
Q3(11.1–12)	103	60.9	144	47.5	2.0	1.1	3.7	0.043
N.I.	2	1.2	17	5.6				
Type of disinfectant								
Non-Guanidine	5	3.0	31	10.2	1.0	Ref		
Guanidine	161	95.3	264	87.1	2.6	0.9	8.0	NS
N.I.	3	1.8	8	2.6				
No of disinfectant products used								
1	95	56.2	179.0	59.1	1.0	Ref		
2	53	31.4	90.0	29.7	1.2	0.7	2.1	
3	18	10.7	25.0	8.3	2.2	0.9	5.2	0.086
N.I.	3	1.8	7	2.3				
Average distance of the bed apart from humidifier, meter								
> 1m	114	67.5	256	84.5	1.0	Ref		
0.5–1 m	42	24.9	42	13.9	2.7	1.5	5.1	
< 0.5 m	10	5.9	4	1.3	13.2	2.4	73.0	<0.0001
N.I.	3	1.8	1	0.3				
Cumulative use hours, hours ^a^								
Q1(<1260)	32	18.9	84	27.7	1.0	Ref		
Q2(1261–2352)	46	27.2	70	23.1	1.4	0.7	2.8	
Q3(2353–5040)	43	25.4	72	23.8	1.6	0.8	3.2	
Q4(5041–39116)	45	26.6	75	24.8	1.5	0.7	2.9	0.27
N.I.	3	1.8	2	0.7				
Airborne disinfectant intensity, *μg*/m^3^, ^b^								
Q1(<317.1)	35	20.7	77	25.4	1.0	Ref		
Q2(317.2–508.5)	36	21.3	76	25.1	1.0	0.5	1.9	
Q3(508.6–942.5)	44	26.0	67	22.1	1.2	0.6	2.4	
Q4(942.6–4946.9)	50	29.6	63	20.8	2.6	1.2	5.3	0.009
N.I.	4	2.4	10	3.3				
Cumulative exposure level, unit-less, ^c^								
Q1(<512000)	33	19.5	76	25.1	1.0	Ref		
Q2(512001–1464515)	45	26.6	69	22.8	1.1	0.6	2.3	
Q3(1464516–3928750)	36	21.3	75	24.8	0.9	0.5	1.9	
Q4(3928751-1x10^8^)	51	30.2	61	20.1	2.0	1.0	4.1	0.071
N.I.	4	2.4	22	7.3				

Note: HDLI: Humidifier disinfectant-associated lung injury. N.I: No information

^a^ Cumulative disinfectant use hours were calculated by multiplying together the total years, months per year, weeks per month, days per week, and hours per day.

^b^ Airborne disinfectant intensity was calculated by multiplying together the bulk level of humidifier disinfectant identified in the specified disinfectant brand (*μg*/mℓ), the average volume (mℓ) added to humidifier, and the room volume (area x height, m^3^).

^c^ Cumulative exposure level = a*b.

ORs and CIs were estimated by unconditional regression, adjusted for stratification variables, age, sex, no of chemical used in house, presence of factory around house and age group.

## Discussion

Our study found several humidifier disinfectant exposure-related variables and demographic characteristics that significantly increased the risk of HDLI in a family-based study. In particular, disinfectant-related metrics were found to be significantly associated with the risk of HDLI in a dose-response manner.

First, airborne disinfectant exposure metrics providing a measure of average patterns, including intensity, average hours sleeping in a room with an operating humidifier treated with disinfectant per day and average distance of the bed from humidifier, showed significant dose-response associations with the development of humidifier disinfectant associated HDLI. Cumulative disinfectant inhalation exposure level was also significantly associated with the risk of HDLI with marginal *P* trend (p = 0.070) (Table 3). In contrast, other metrics regarded as providing a measure of chronic exposure, including total humidifier disinfectant use years, humidifier disinfectant use months and cumulative humidifier disinfectant use hours, were not related to the risk of HDLI. These differences by exposure metric may be because humidifier disinfectants were used in a variety of patterns, such as intermittently during specific events or seasons or times or continuous use, which may influence the risk of HDLI. Only four study subjects (HDLI patient = 1, non-patient within family = 3) used humidifier disinfectant continuously whereas the remainder of the study subjects were found to run their humidifier with disinfectant according to diverse interval durations. Unfortunately, we were unable to examine how different patterns of humidifier disinfectant use were associated with HDLI risk.

Second, “type of disinfectant” and “number of humidifier disinfectant brands used” showed a marginally significant association with the risk of HDLI. Those using humidifier brands containing the guanidine disinfectant chemical group (adjusted OR = 2.6, 95% CI = 0.9–8.0) were evaluated to be at higher HDLI risk than those using brands containing the non-guanidine group. In addition, the highest number of humidifier disinfectant brands used (n ≥ 3) (adjusted OR = 2.2, 95% CI = 0.9–5.2) increased HDLI risk approximately two-fold compared with the lowest quartile (only one type of disinfectant product used). The association of guanidine disinfectant chemicals with the risk of lung diseases including HDLI in South Korea was previously reported by Park, Leem [15], who found that 38 self-reported lung disease patients from 17 families with a minimum of two patients responded that they used humidifier disinfectant brands containing the guanidine chemical group (PHMG = 36, or PGH = 2) [15]. For HDLI patients who used both humidifier disinfectant brands containing guanidine and those with non-guanidine disinfectant, there was insufficient numbers to examine whether the type substantially contributed to the development of HDLI or whether a mixed or interaction effect of the two types exists.

Lastly, the two susceptible groups, pregnant women and pre-school children ≤ 6 years old accounted for 82.2% (n = 139) of HDLI patients in this study and were at an increased risk of HDLI compared to 7- to 18-year-olds and > 18 year-olds (Table 1). The increased risk in these two groups may represent a reporting bias, where lung injuries may have been more likely reported for the very young and for pregnant women because of their increased concerns with health effects. Seventy-eight (80%) of pre-school children cases were within the toddler period (ages 1 to 3 years) and 24 (23%) were infants of between birth to 12 months. We did not find any significant factors indicating increased use of disinfectant for younger children and pregnant women under the assumption that they would be more susceptible to molds that might occur without disinfection. In an analysis where HDLI cases ≤ 6 years old were categorized into either one- or two-year intervals, the average total volume added to humidifier per day, average frequency of addition to humidifier per day, average distance of the bed from the humidifier and average airborne disinfectant level (*μg*/m^3^) were all found to be not significantly higher than in the case of the other three age groups. Therefore, we assumed that the following two factors may be associated with the high incidence rate of HDLI for pre-school age groups. Firstly, these toddler groups spend most of their time at home with their mother with a humidifier using disinfectants. We found that those two patient groups used humidifier disinfectant intensively during the perinatal and/or early infancy period when pulmonary development has not been completed [12]. Secondly, these periods may have caused increased susceptibility to HDLI [16]. In particular, children differ substantially from adults in terms of exposure dose due to tidal volume (greater intake of air), physiological factors (greater circulatory flow rates), pharmacokinetics (respiratory absorption rates) and pharmacodynamics (immature host defenses) [17, 18], all of which may be closely associated with the higher incidence rate of HDLI cases among pre-school children. In addition, hormonal effects in pregnant women leave them more vulnerable to toxic inhalation injury resulting from increases in ventilation, tidal volume, and minute ventilation [19]. And gradual increases in blood volume during gestation may exaggerate the influence of toxic chemical mediators in pregnant women [20]. The dose-response relationship we found in several humidifier disinfectant use variables still remains significant after several age classifications were adjusted, even if there is a change in statistical p-values.

An increased risk of HDLI with aerosolized humidifier disinfectant use is biologically plausible based on the ingredients of humidifier disinfectant products containing guanidine and non-guanidine chemicals groups and the ultrafine particle size of dispersed disinfectant. Since the identification of humidifier disinfectant being associated with lung injury, two toxicological studies were conducted to examine the toxicological effect of PHMG and PGH, the major components of humidifier disinfectant in South Korea. In a histopathological study using rats, the findings for products containing PHMG and PGH were identical to those regarding the patients with lung injury [21]. Kim, Choi [22] observed granulomatous obliterative bronchiolitis (OB), bronchitis, collagenized fibrosis, alveolar bronchiolarization, and extensive squamous metaplasia in rats exposed to humidifier products with PHMG and PGH at concentrations of 0.4 mg/m^3^ and 1.75 mg/m^3^ for ten and seven weeks, respectively.

The inhalation route predominates because the disinfectant dissolved in humidifier water was dispersed into the air by the humidifier’s aerosolizer. The ultrasonic humidifiers used by most patients readily disperse aerosol water droplets ranging in size from 0.5 to 3 μm, which easily reach the distal airways [23, 24]. The amount of inhaled disinfectant is expected to be higher the closer one is to the humidifier, which is supported by the increasing HDLI risk with humidifier proximity observed here (< 0.5 m; adjusted OR = 13.2, 95% CI = 2.4–73.0, 0.5–1 m; adjusted OR = 2.7, 95% CI = 1.5–5.1), compared with those who remained farther than 1 m away (reference).

To our knowledge, this is the first study not only to identify specific disinfectant-exposure factors influencing the risk of HDLI, but also to examine dose-response relationship between exposure to humidifier disinfectant and HDLI risk. The overall strengths of our study are the population-based design with HDLI cases clinically examined by a team of 12 pediatric pulmonologists, radiologists and pathologists, as well as extensive interviews allowing for the evaluation of possible confounding by hypothesized HDLI risk factors, and assessment of humidifier disinfectant exposures that extend years before diagnosis and encompass other hazardous agent exposures including household chemicals and the presence of hazardous facilities near the home. In particular, there was limited knowledge regarding humidifier disinfectant product formulations used in the past, the potential exposure levels experienced, and the biological activity and toxicity of several disinfectant chemical constituents alone and in combination. We developed a systematic and transparent retrospective exposure assessment strategy [12]. The responses of study subjects to questions related to disinfectant use characteristics and our field investigation results were directly used to develop measures of typical and chronic disinfectant exposure. We aimed to improve the accuracy of the participants’ recall by showing photographs of each disinfectant brand and asking additional questions that may be specific to respective disinfectant types [12].

An additional strength was the control group of non-patient family members who lived in the same residence as HDLI patients, who were chosen to minimize reporting bias because they would be similarly aware of the potential health effects related to humidifier disinfectants. In contrast, HDLI cases and population controls may have differential recall and awareness of the study hypothesis. Werler, Shapiro [25] hypothesized that this type of bias occurs when cases are aware of the study hypothesis, resulting in higher exposure reporting and, consequently, an elevated odds ratio [25]. Here, the non-lung injury familial group showed similar response patterns as the HDLI family group because they lived together, although within-family differences in humidifier use were observed and captured in our metrics. Our use of non-lung patients of familial as a control group is also supported by the results reported by Milne, John [10], who found that several key associations were not evident when cases were compared with population controls, but were observed when cases were compared with sister controls who lived in the same house as breast cancers cases. Another limitation of this study is the likelihood of selection bias from the sampling approach based on people who self-reported to the KCDC. There could be also limitation as to how well this might represent the broader population of HDLI cases or who was not included in the study. A national surveillance program should be designed not only to examine how HDLI progresses into further chronic health effects such as cancer or decrease of lung function, but also to identify new HDLI cases due to the use of humidifier disinfectant. Recently, the 2^nd^ round of the investigation has been operated to officially collect HDLI cases who might not registered in the 1^st^ round. Examining the mechanism of how humidifier disinfectant eventually results in HDLI, including interstitial lung disease, is challenging since there is currently only limited knowledge regarding the potential exposure levels experienced by different age groups of victims with a wide range of susceptibilities, as well as on the biological activity and toxicity of several disinfectant chemical constituents, both alone and in combination [12].

In conclusion, the use of household humidifier disinfectant contributes to HDLI risk in a dose-response manner. In particular, HDLI risk was elevated among susceptible individuals including pregnant women and the pre-school age group, who tended to have the highest humidifier disinfectant use. Airborne disinfectant intensity, hours sleeping in a room with an operating humidifier treated with disinfectant and average distance of the bed from a treated humidifier were found to be major factors increasing HDLI risk.
